# Impact of Visceral Obesity on Clinical Outcome and Quality of Life for Patients with Multiple Myeloma: A Secondary Data Analysis of STaMINA (BMT CTN 0702) Trial

**DOI:** 10.21203/rs.3.rs-3318127/v1

**Published:** 2023-09-21

**Authors:** Ehsan Malek, Jeries Kort, Leland Metheny, Pingfu Fu, Parameswaran Hari, Gen Li, Yvonne Efebera, Natalie Callander, Muzaffar Qazilbash, Sergio Giralt, Amrita Krishnan, Edward Stadtmauer, Hillard Lazarus

**Affiliations:** University Hospitals Cleveland Medical Center, Case Western Reserve University; University Hospitals Cleveland Medical Center, Case Western Reserve University; University Hospitals Cleveland Medical Center and Case Western Reserve University; Medical College of Wisconsin; Case Western Reserve University; The Ohio State University; University of Wisconisn; The University of Texas MD Anderson Cancer Center; Memorial Sloan-Kettering Cancer Center.; City of Hope; Abramson Cancer Center; Case Western Reserve University

**Keywords:** Multiple myeloma, hematopoietic cell transplantation, obesity, quality of life

## Abstract

Obesity is a common health problem among multiple myeloma (MM) patients, and it has been linked to poor clinical outcomes and quality of life (QOL). We conducted a secondary analysis of the BMT CTN 0702, a randomized, controlled trial comparing outcomes of three treatment interventions after a single hematopoietic cell transplant (HCT), to investigate the impact of visceral obesity, as measured by waist-to-hip ratio (WHR), on clinical outcomes and QOL in MM patients. 549 MM patients, median age 55.5 years, were enrolled in the study. The majority of patients received triple-drug antimyeloma initial therapy before enrollment, and 29% had high-risk disease according to cytogenetic assessment. The median follow-up time was six years. There was no significant association between WHR and progression-free survival (PFS) or overall survival (OS) in MM patients undergoing HCT. Similarly, body mass index (BMI) did not significantly predict PFS or OS. Furthermore, there was no significant correlation between WHR and QOL measures. In conclusion, this study suggests that visceral obesity, as measured by WHR, may not significantly impact clinical outcomes in MM patients undergoing HCT. Further studies utilizing imaging technologies to assess the impact of visceral obesity distribution are warranted.

## INTRODUCTION

Obesity is a growing global health concern, with increasing evidence linking it to an elevated risk of various cancer types, including plasma cell dyscrasia (PCD) [1–10]. The International Agency for Research on Cancer (IARC) considered obesity to be a “preventative factor” for MM [11]. However, conflicting evidence exists regarding the impact of obesity on hematopoietic cell transplant (HCT) outcomes. Some studies have shown a correlation between obesity and worse outcomes, while others have not found any significant association [12]. Vogl et al. [13] reported data on 1,087 multiple myeloma (MM) patients who received high-dose melphalan with or without total body irradiation (TBI) as a conditioning regimen for autologous HCT, and found no indication of worse outcomes for subjects with higher body mass index (BMI)s. Patients receiving high-dose melphalan alone had similar treatment outcomes across different BMI groups. However, among patients who received conditioning with both melphalan and TBI, those who were obese (BMI 30–34.9) or severely obese (BMI > = 35) had lower rates of relapse and superior PFS and OS compared to those who were normal weight (BMI 20–24.9).

Obesity is associated with a pro-inflammatory state and increase in circulating concentrations of cytokines, including insulin-like growth factor-1, hepcidin, tumor necrosis factor-alpha and interleukin-6 (IL6); such are considered crucial to the pathogenesis of MM [14, 15]. Adipose tissue is principally deposited in two major compartments - subcutaneously and visceral. It is thought that visceral adipose tissue (VAT) is more metabolically active than peripheral subcutaneous adipose tissue (SAT) [16–18]. Visceral obesity is more strongly associated with increased risk of insulin resistance, the metabolic syndrome and cardiovascular diseases than BMI alone [19]. These systemic effects exerted by visceral adiposity are also involved in the genesis of cancer [20]. SAT, on the other hand, frequently has a somewhat opposite metabolic impact, as SAT expansion ameliorates insulin sensitivity and decreases type 2 diabetes. The interplay of excess VAT and clinical outcome for pts with PCD has not been delineated before, mostly due to lack of an accurate assessment tool for VAT.

BMI has traditionally been used as the primary tool for assessing obesity, but it does not take into account the differences in size and body fat composition and is influenced by various factors such as ethnicity and age. This limitation can be noteworthy in an obesity-associated cancer with a strong racial and age preference such as MM. Waist-hip ratio (WHR), on the other hand, may be a more accurate indicator of visceral obesity and has been shown to be a better predictor of metabolic risk and cardiovascular disease than BMI [19].

In addition to the known adverse health effects of obesity and the increased risk for comorbidities, this condition may alter chemotherapy dosing and pharmacokinetics that can affect patient outcomes [21]. Most studies that associate obesity with outcome have been limited by retrospective nature, the inclusion of multiple diseases, different conditioning regimens, and the use of body surface area as a surrogate of obesity.

The Blood and Marrow Transplant Clinical Trials Network (BMT CTN) conducted the phase 3 0702 (ClinicalTrials.gov) STaMINA trial which compared a single autologous HCT with or without consolidation therapy, versus tandem autologous HCT with lenalidomide maintenance therapy for MM patients [22]. We conducted a companion prospective evaluation in these patients in order to examine the role of visceral obesity in patient outcomes. In addition to BMI, we incorporated the simple anthropomorphic measurement of waist-hip ratio (WHR) [23, 24] to assess the impact of visceral obesity. Herein, we correlate and report these measurements with patient outcomes.

## METHODS

### Study Design

The BMT CTN 0702 (STaMINA) prospective, phase III randomized trial enrolled 758 MM patients comparing progression-free survival (PFS) and overall survival (OS) for three, randomized, therapeutic interventions undertaken after a single autologous HCT: (1) a second HCT followed by lenalidomide maintenance therapy (n = 247); (2) consolidation with lenalidomide/bortezomib/dexamethasone therapy then followed by lenalidomide maintenance (n = 254); or (3) lenalidomide maintenance therapy alone (n = 257). The study enrolled patients from June 2010 through November 2013; the primary analysis revealed that all three arms had similar clinical outcomes, including quality of life (QOL) scores using the SF-36 and FACT-BMT instruments which were reported previously [25]. The objective of this ancillary study was to assess impact of visceral obesity measured by WHR on clinical outcomes as well as QOL.

### High-dose melphalan dosing

Melphalan dosing was standardized based on patient weight, with ideal body weight (IBW) used for patients weighing between 100–120% of their IBW. Actual body weight (ABW) was used for patients weighing less than 100% of their IBW, while adjusted ideal body weight (AIBW) was used for those subjects weighing over 120% of their IBW, with a 25% dose modification.

### Anthropomorphic Measurement

Patients were measured at three time points during the first year of the BMT CTN 0702 trial: baseline, prior to maintenance therapy and at one year after randomization to BMT CTN 0702. Patients who discontinued protocol-specified therapy for any reason were not required to have subsequent anthropomorphic measurements taken.

Assessment of anthropomorphic measurements were standardized among participants. Measurements were taken by medical personnel assigned by each institution in accordance with standardized techniques distributed to all participating sites. A special video was made and distributed among participating centers to illustrate how to execute this measurement and facilitate uniformity and reproducibility.

Height and weight were measured without shoes and in light clothing or hospital gown. Self-reported height was acceptable only if the patient was unable to stand.


$$\text{Body}\;\text{mass}\;\text{index}\;\text{(BMI)}=\text{weight}\;\text{(kg)}\;/\;\text{height}\;{\left(\text{m}\right)}^{2}$$

Waist and hip measurements were taken in underwear or with light clothing or hospital gown and were recorded in centimeters. The waist measure was taken at the point mid-way between the lower ribs and the iliac crest, with the patient standing and exhaling gently. The hip measure was taken at the widest hip circumference with the patient standing.

We used the cutoff of WHR > = 0.9 and BMI > = 25 to define obesity [26]

### Patient-reported outcomes

English and Spanish speaking patients completed the SF-36 and FACT-BMT instruments which were self-administered. Questionnaires were administered after randomization to treatment arm and annually until 4 years post-randomization. The FACT-BMT version 4.0 instrument is comprised of a general core questionnaire, the FACT-G that evaluates the health-related quality of life (HQL) of patients receiving treatment for cancer, and a specific module, BMT Concerns, that address disease and treatment-related questions specific to HCT. The FACT-G consists of four subscales developed and normalized in cancer patients: Physical Well-being (PWB), Social/Family Well-being (SWB), Emotional Wellbeing (EWB), and Functional Well-being (FWB) subscales were positively scored, with higher scores indicating better functioning. The FACT-BMT Total, the grand total of all items in the FACT-G and BMT modules, was used as the outcome measure in summarizing the FACT-BMT data. A change in 2–3 points is considered a minimal clinically important difference on the FACT-BMT [27].

### Statistical Analysis

The study design, sample size estimation and trial details were described previously [22]. The primary objective was to examine the effect of BMI and waist-hip ratio (WHR) on QOL. All patients with available WHR measurement were included in this study (N = 549). QOL was assessed along with WHR at baseline, 1-year, 2-year, 3-year, and 4-year time points. Time-to-event end points were estimated from the time of random assignment. In this project, we focused on five major components of QOL mentioned in the section of QOL assessment. The temporal profiles of QOL were visualized using a scatter plot. In order to estimate the effect of BMI and WHR on QOL, a mixed-effect model approach was used. In this mixed-effect model, we assumed the measurements of QOL during follow-up from the same patient were correlated and unstructured covariance was used for inference. The selection of covariates in the final multivariable mixed-effect model was based on Akaike information criterion (AIC). Chi-squared test was used to estimate the association between categorical variables and WHR (low vs high, cutoff point = 0.9) and t-test was used to detect the difference of continuous measurements between WHR groups (low vs high, cutoff point = 25). OS and PFS were analyzed using Kaplan-Meier method with log-rank test and Cox model. All tests were two-sided and p-value < 0.05 were considered statistically significant.

## RESULTS

### Patients’ characteristics

**Figure 1:**
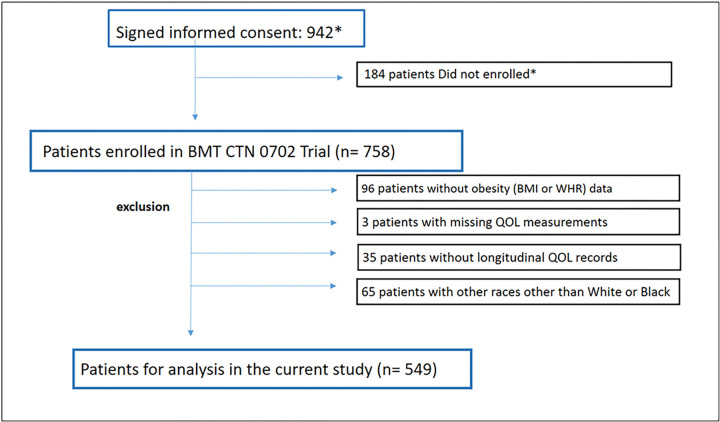
CONSORT diagram. (*) Sites were not required to register patients who signed informed consent but did not enroll. BMI: Body Mass Index, WHR: Waist-Hip Ratio, QOL: Quality of Life

From June 2010 through November 2013, 758 patients were enrolled on BMTCTN 0702 clinical trial. Median age at enrollment was 55.5 years and 17% were African American. Median follow up time was 6 years. Data from a total of 758 patients was used in this study before quality control. Patients with missing obesity information (N = 96), missing QOL measuring (N = 3), unavailable longitudinal QOL records (N = 35) and other missing variables (N = 65) were excluded (Figure-1). Finally, 549 patients (72%) formed the study cohort. More White patients (N = 446) had high WHR compared to African American patients (N = 103) (p = 0.026), and more male patients (N = 332) were enrolled with high WHR compared to female patients (N = 217) (p = 0.001). Before enrollment, 73% of patients initially received triple-drug antimyeloma initial therapy. Lenalidomide, bortezomib and dexamethasone (RVD) was used as initial therapy in 55% of patients while bortezomib, cyclophosphamide, and dexamethasone (VCD) was used in 14% of patients. The median time from initiating therapy to enrollment was 5.2 (range, 2.1 to 14.4) months. At enrollment, 91% of patients had a partial response or better, 47% had at least a very good partial response, and 18% attained complete remission (CR or stringent CR). High-risk MM was defined by presence of high b2-microglobulin ( > = 5.5 mg/L) or presence of cytogenetic abnormalities, including t(4;14), t(14;20), t(14;16), deletion (17p) detected by fluorescence in situ hybridization [28], 29% of patients were classified as having high-risk disease.

### Correlation of BMI/WHR:

**Figure 2:**
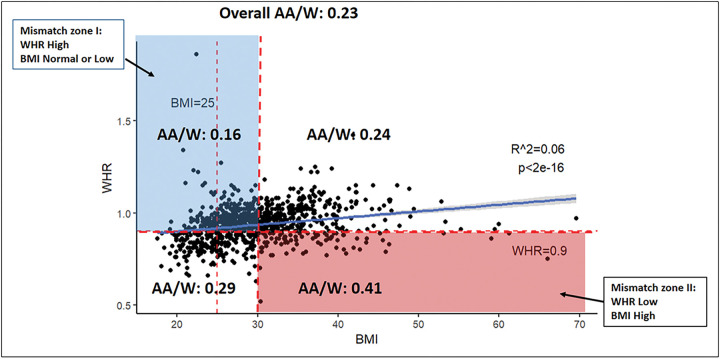
Scatter plot of BMI vs. WHR. Two mismatched between BMI and WHR were recognized. Patients with high WHR but normal/low BMI, or low WHR but with high BMI were characterized as zone I (blue) and II (red), respectively. WHR: Waist-to-Hip ratio, BMI: Body Mass Index

Median BMI was 29.11 (range, 17.58 to 69.59) and median WHR was 0.94 (range, 0.52 to 1.86). A total of 438 (79.8%) patients had BMI > 25 and 111 (20.2%) were ≤ 25. Further, 362 (66%) patients had WHR > 0.9 and 187 (34%) patients had WHR ≤ 0.9. Although BMI and WHR were matched in large portion of patients, i.e., both were high or low, but two mismatched zone I and II between BMI and WHR were recognized (Figure-2). These mismatch zones, or discrepancies between BMI and WHR, represent patients at risk where BMI may not be the optimal tool for body content assessment. The African American to White patients was associated with high BMI/low WHR, p-value: 0.044 (Figure-2, Sup Table-1). Age was not associated with mismatched zone (p-0.446).

### Survival based on binary and continuous classifications:

Binary WHR (> 0.9 and ≤ 0.9) did not significantly predict PFS, (HR 0.95, and 95% CI 0.604–1.819, p: 0.87), nor OS (HR 1.14, and 95% CI 0.833–1.572, p: 0.41). Similarly, BMI (> 25 and ≤ 25) was not significantly associated with PFS (HR 0.89, and 95% CI 0.667–1.20, p: 0.46), or OS (HR 1.05, and 95% CI 0.604–1.817, p: 0.87).

### Quality of life:

**Figure 4:**
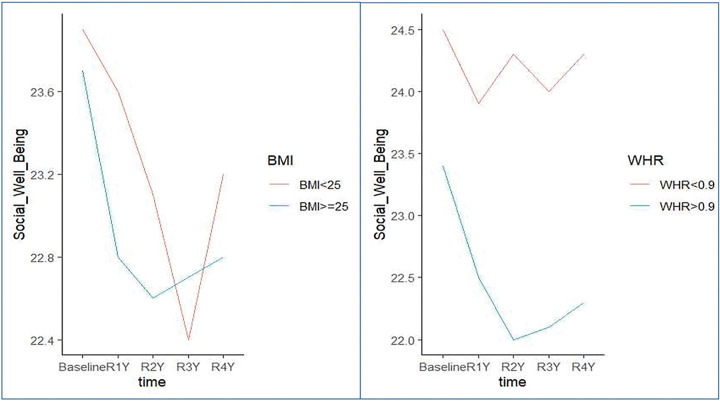
Social Well-being Score based on BMI and WHR

When comparing the QOL measures over time, SWB was the only QOL tool that was consistently higher in patients with low compared to high WHR (baseline: 24.5 vs. 23.4 P = 0.002; year 1: P = 0.002; year 2: P = 0.001; at year 3: P = 0.001, year 4:P = 0.006) (Figure-4). WHR could not predict for other 4 QOL tools: **PWB** (baseline: 20.3 vs. 20.0 P = 0.6; year 1: P = 0.8; year 2: P = 0.4; at year 3: P > 0.9, year 4:P > 0.9), **EWB** (baseline: 19.0 vs. 19.0 P = 0.9; year 1: P = 0.9; year 2: P = 0.3; at year 3: P = 0.4, year 4:P = 0. 6), **FWB** (baseline: 17.6 vs. 16.8 P = 0. 2; year 1: P = 0.2; year 2: P = 0.12; at year 3: P = 0.2, year 4:P = 0.2), **BMTS** (baseline: 28.5 vs. 28.6 P = 0.8; year 1: P = 0.3; year 2: P = 0.5; at year 3: P = 0.7, year 4:P = 0.3). In our mixed model, WHR neither as a binary variable nor as continuous variable was significantly associated with overall QOL outcomes after adjusting for race, gender, age, KPS score, and regimen (Figure-2).

## DISCUSSION

This prospective study aimed to assess the impact of visceral obesity on clinical outcomes and QOL in MM patients enrolled in the BMT CTN 0702 trial. The results showed that visceral obesity, as measured by WHR, was not associated with any significant difference in clinical outcomes or QOL in MM patients who underwent HCT. Importantly, more African-Americans had higher BMI but low WHR in comparison to White patients but there was no difference in outcomes based on race or age. Given a high number of enrolled patients resulting in relatively low type II error and high power to detect a difference in outcome based on visceral obesity, the negative result of our study can be informative. Furthermore, our finding suggests obesity should not count as an important decision making factor when it comes to HCT eligibility for MM. Hence, commonly used Comorbidity indices such as Sorror score [29] that includes obesity as important prognostic factor, should not be extrapolated for MM patients.

Trajectories of quality-of-life recovery and symptom burden after HCT from this trial were reported previously [25]. Survivors of MM who achieve disease control after HCT experience a significant recovery in FACT-BMT and subscale scores, with scores returning to population norms within one year of HCT. However, many patients still report moderate to severe symptoms at the one-year mark and beyond, including frequent infections, and gastrointestinal and skin problems. In this study, we investigated whether obesity assessed by BMI either WHR would predict worse QOL, but we found no significant association between neither, whether as a binary or continuous variable. Further, overall QOL outcomes after adjusting for factors such as race, gender, age, KPS score, and treatment regimen did not differ.

Interpreting reports of obese patients undergoing HCT is challenging due to variations in obesity definitions, underlying disease and chemotherapy preparative regimens used. Additionally, the lack of a consistent standard for calculating chemotherapy doses in this population makes it challenging to interpret these findings. Future studies using specific regimens in this population are urgently needed [12].

Our study provides a prospective approach, which allowed for accurate measurement of anthropomorphic variables and minimized the risk of race- or age-related biases. The standardized techniques for measuring anthropomorphic variables across all participating sites further enhance the feasibility of acquiring WHR and ensuring reliability of the data. Melphalan dosing was also standardized and uniform in this study based on patient weight.

WHR is emerging as a strong tool enabling practitioners to assess cardiometabolic risk associated with increased adiposity in adults. Unequivocal evidence supporting its superiority to BMI lead to recommendations regarding this measurement as ‘vital signs’ in routine clinical practice [30]. STaMINA trial utilized the measurement for the first time in the field as a better indicator for visceral fat. Despite that, our study didn’t show a clinical difference in outcomes using this measure.

Although we used a valid as well as feasible method to assess fat distribution, WHR probably does not capture a full topography of adipose tissue throughout body. Modern imaging technology provides even more accurate approaches to quantitate body fat and characterize its distribution. Total body dual energy X-ray absorptiometry (DEXA) scans, often employed for diagnosing osteoporosis or osteopenia, can be used to also distinguish fat mass and lean body mass, assess regional distribution and provide quantitative estimates of components and total adiposity. Other techniques, such as magnetic resonance imaging (MRI), now are also used to provide images to quantitate visceral and subcutaneous adipose tissue volume and ectopic fat [31, 32]. These technologies provide high dimensional data on fat composition (e.g., three-dimensional volume of visceral, abdominal subcutaneous and gluteofemoral adipose tissue) that can be utilized to train machine learning models predicting health risks associated with different levels and patterns of adiposity [33, 34].

Despite these strengths, there are limitations to this study that should be acknowledged. First, although our data were collected prospectively, this approach was a secondary data analysis of the BMT CTN 0702 trial, which was not designed specifically to assess the impact of obesity on MM outcomes. Second, the follow-up period for this study was relatively short, which may have limited the ability to detect differences in long-term outcomes. Finally, there might be an inherent selection bias as patients enrolled on a clinical trial generally have better functional and socioeconomic status and easier access to anti-cancer therapies at an earlier stage [35]. These factors could potentially jeopardize the generalizability of the negative finding of this trial.

In conclusion, this study suggests that visceral obesity, as measured by WHR, is not associated with any significant difference in clinical outcomes or QOL in MM patients who underwent HCT. While the use of WHR is a significant improvement over previous studies that relied upon BMI alone, we could not detect a clinically meaningful difference between using BMI and WHR. Further studies are needed to confirm these findings and to assess the impact of visceral obesity using modern imaging technologies on the outcomes of MM patients undergoing other treatments or in different stages of the disease.

## Figures and Tables

**Figure 3:**
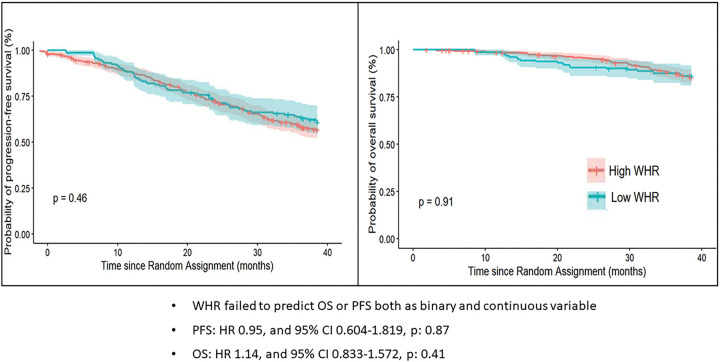
PFS and OS based on binary classification.
